# Dissecting Virus Infectious Cycles by Cryo-Electron Microscopy

**DOI:** 10.1371/journal.ppat.1005625

**Published:** 2016-06-30

**Authors:** Kelly K. Lee, Long Gui

**Affiliations:** 1 Department of Medicinal Chemistry, University of Washington, Seattle, Washington, United States of America; 2 Biological Structure Physics and Design Program, University of Washington, Seattle, Washington, United States of America; University of Michigan Medical School, UNITED STATES

In response to events such as receptor binding and endocytic triggers, viruses undergo large-scale, dynamic conformational changes necessary for cell entry and genome delivery. In later stages of the infectious cycle, replication machinery must read and synthesize nucleic acid strands to generate new copies of genetic material, and structural proteins must assemble and package the appropriate contents in order to produce new infectious particles. Structural elucidation of these events is key to understanding them and their inhibition by antiviral agents such as neutralizing antibodies and drugs. Electron microscopy is a versatile technique that offers the ability to resolve three-dimensional structures of individual viral proteins and whole virions in multiple functional states, even in cells at different stages of infection (Fig 1). Here we focus on the use of transmission electron microscopy of frozen-hydrated specimens, i.e., cryo-electron microscopy (cryo-EM). A major advantage of cryo-EM over other structural approaches is that samples of a broad range of sizes can be imaged under near-physiological conditions with native hydration intact. This approach does not require the specimen to be fixed, stained, or coaxed into a crystalline lattice. This versatility has enabled cryo-EM to expand the envelope of structural virology and opened new avenues for understanding the molecular and cellular processes of virus infection and pathogenesis.

**Fig 1:**
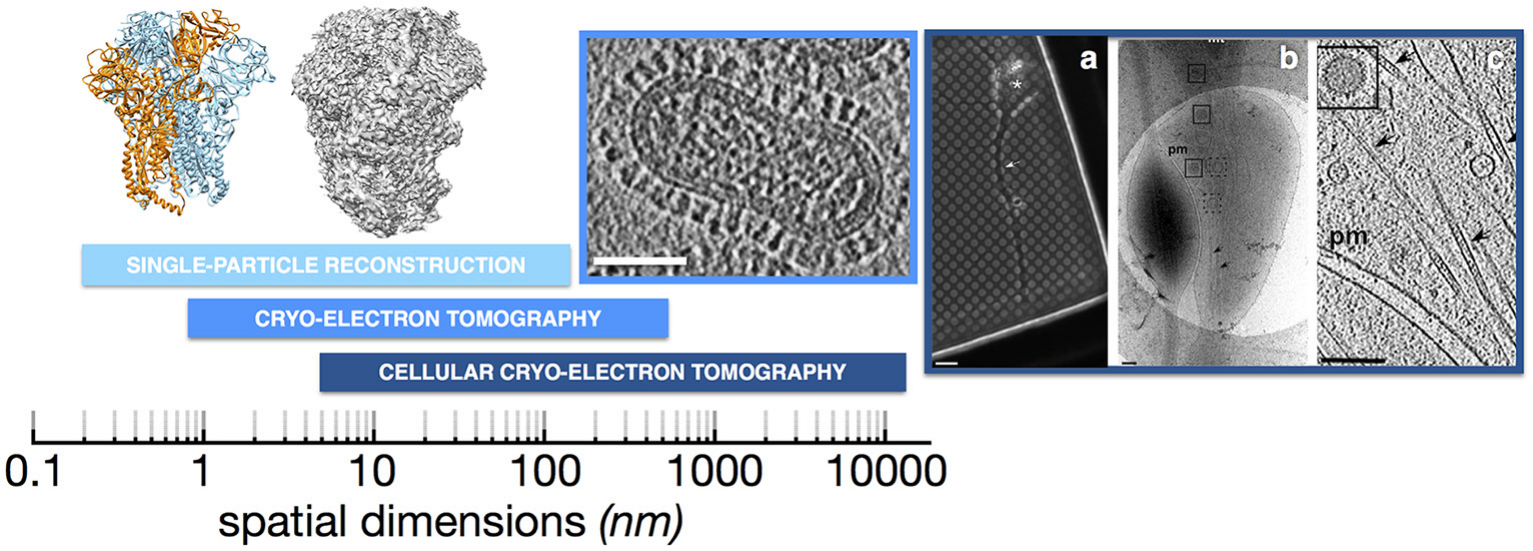
Cryo-electron microscopy (cryo-EM) enables one to gain insight into viruses over many levels of structural hierarchy, ranging from determination of near-atomic resolution structures of subunits and symmetrical virus assemblies to ultrastructural analysis of virus-infected cells. **Left**: Single-particle reconstructions have reached near-atomic resolution, enabling the folds of viral proteins to be determined. The structure of a mouse coronavirus S glycoprotein spike was determined to 4.0 Å resolution by single-particle cryo-EM. A combination of homology modeling and de novo polypeptide chain modeling was used to determine the protein structure (one subunit in the homotrimer is rendered orange; pdb3JCL.ent [11]). The electron density map is shown to the right of the ribbon diagram (EMD-6526 [11]). **Middle**: Cryo-electron tomography (Cryo-ET) can be used to image the three-dimensional structure of non-symmetrical viruses such as influenza A virus shown here ([49]; scale bar 50 nm). **Right**: Cryo-ET is also being used to image viruses in the context of intact cells as in this example that examined HSV-1 transport along an axon of an intact neuron [55]; a) shows the neuron grown on an EM grid that is punctuated by 2 μm holes in the carbon film, b) shows a close-up view of a portion of the axon that spans across a hole, and c) shows the reconstructed cryo-electron tomogram with an HSV-1 core particle highlighted by the box. (Scale bars: A 6 μm; B,C 200 nm). Taken together, these types of cryo-EM-based structural approaches are bringing viral infectious cycles into clearer view.

In this micro-review, we examine general approaches and recent applications of cryo-EM to virology. The reader is referred to a number of excellent reviews for more in-depth discussion of current methodology (e.g., [1,2]). Electron microscopy and structural virology have a long, shared history dating to the first images of bacteriophages gathered using some of the earliest electron microscopes [3]. As the technique of cryogenically preserving unfixed samples was being developed, again, viruses were among the first test subjects that helped to demonstrate the utility of cryo-EM for characterizing structure of large biological assemblies [4–6].

## Single-Particle Reconstruction of Viral Components and Virus Particles

Single-particle cryo-EM reconstruction is typically applied to purified proteins and macromolecular complexes with the aim of resolving the organization of multi-component assemblies. Until recently, this approach offered primarily domain- or subunit-level structural detail. Even at this resolution, significant information about virus structure and conformational changes is uniquely accessible by cryo-EM. Whereas crystallographic studies of viruses have been most effective in characterizing highly stable forms of viruses and their capsids, with cryo-EM, viruses can readily be examined in different structural states under a range of conditions. This versatility is exemplified by studies of dengue viruses in immature and mature forms [7]. More recently, comparisons of this insect-borne pathogen’s structure at the physiological temperatures of both mosquito vector and human host revealed a large-scale, temperature-dependent conformational change with differences in E protein subunit organization and exposure of the underlying viral membrane [8]. EM studies such as these have deepened our understanding of how a virus can both maintain a robust assembly that protects the genome in the vector and outside the human host, and then transform into a state that is primed for entry and uncoating once a susceptible host cell is encountered.

Powerful, new direct electron detectors that gather images with greater sensitivity and with the ability to correct for image blurring due to minute sample movement [9], coupled with high-throughput data collection [10] and advanced methods for processing reconstructions [1], have led to breakthroughs in resolution of single-particle reconstructions such that polypeptide chains can be traced de novo (e.g., [11,12]), and amino acid types can be identified from the electron density of their sidechains (e.g., [13]). Near-atomic resolution structures have been determined by single-particle cryo-EM for icosahedrally symmetrical viruses as diverse as flaviviruses, including dengue [14] and the emergent Zika virus [15], dsDNA viruses such as adenovirus [16] and a thermophilic virus that exists in the hot springs of Yellowstone National Park [17], and the dsRNA rotavirus [18], to name a just a few examples. These detailed structures provide insight into residue-level interactions that stabilize capsid assemblies. For surface and capsid proteins that are targets for neutralizing antibodies, by enabling Fab-virus complexes to be studied in a fairly straightforward manner, cryo-EM analysis is proving exceptionally useful for discerning the full epitopes that are recognized by antibodies, including, in some cases, portions of glycan in addition to protein features (e.g., [19–21]).

Highly symmetrical subassemblies, such as bacteriophage portal, tail, and baseplate complexes, have also been characterized in multiple conformational states by single-particle cryo-EM (e.g., [22]). This highlights the ability of cryo-EM to characterize the structural basis of biological function, such as genome delivery, that is carried out by these complex macromolecular machines. Helical structures are also amenable to high-resolution characterization as demonstrated in reconstructions of measles virus nucleocapsids [23] and a rod-shaped hyperthermophilic virus, which for the first time revealed A-form DNA encapsulated within a helical protein sheath [24].

In most cases with symmetrical objects, the underlying symmetry is utilized during image processing to improve the ratio of signal-to-noise and enhance resolution. Beyond their capsids, icosahedral viruses are in fact non-symmetrical due to the incorporation of internal components, such as the nucleic acid genomes and portal complexes located at particular vertices in some bacteriophage and viral capsids. Asymmetric reconstructions can help to identify the location of these subassemblies, as was recently shown to stunning effect in characterization of cytoplasmic polyhedrosis virus (CPV) that identified the organization of genomic segments and transcription enzyme complexes within the icosahedral shell [25].

Thanks in large part to the advances in imaging technology, it is now also possible to tackle structures of viral components that are far smaller than the capsids that were the primary domain of cryo-EM analysis for many years. Near-atomic resolution structures of viral components in the ~400 kDa range, such as polymerase complexes [26] and natively glycosylated surface glycoprotein spikes, are now attainable (Fig 1) [11,19]. Recent single-particle cryo-EM structures of pre-fusion coronavirus S spikes from mouse hemorrhagic virus and a human beta-coronavirus revealed unexpected fusion protein architecture and previously unappreciated structural similarities with paramyxovirus F proteins, suggesting a possible common ancestry [11,12]. In addition, cryo-EM and crystal structures of a soluble, mutation-stabilized form of the HIV-1 Env glycoprotein trimer [27,28] as well as a cryo-EM reconstruction of detergent-extracted native Env [29] have resolved controversies that accompanied initial EM models of this biomedically important cell entry machine and target for neutralizing antibodies [30–33]. Cryo-EM and crystal structures of a retroviral integration complex have also recently demonstrated that the proteins form an octomeric rather than the previously anticipated tetrameric ring structure [34,35]. These examples demonstrate the rapid advances that cryo-EM is making in the characterization of viral machinery under native solution conditions. In many cases, the samples that can be studied are refractory to crystallization, e.g., they bear flexible glycan chains or membrane-associative domains (or both!).

Single-particle cryo-EM reconstruction relies upon imaging thousands and even hundreds of thousands of individual particles in a sample. One caveat to the interpretation of biological data based on cryo-EM structures stems from the fact that, over the course of image processing to generate a three-dimensional structure, particles from the initial dataset that are in similar conformations are grouped and averaged. This often results in the removal of significant populations of particles from the set used to generate the final, refined structure. The end result may be reconstructed from a small subset of the population that was present in the sample. If a sample exhibits compositional heterogeneity, such as with different numbers of ligands bound to an oligomeric viral glycoprotein spike [27], or conformational heterogeneity, such as with movement of domains in a complex, this can confound the connection between the singular structure that is obtained and behavior of the sample population. Fortunately, new analytical tools are increasingly gaining in ability to parse the heterogeneity that exists within even “pure” specimens and more thoroughly analyze variation within the specimen population.

Even with this added capability to parse heterogeneous samples, it is valuable to compare the EM structures against structural information based on the bulk population. Methods such as small-angle X-ray scattering, structural mass spectrometry, or nuclear magnetic resonance spectroscopy (NMR) provide useful complementary insights into whether the high-resolution structures based upon subpopulations are representative of the total population. Validation of structures against biochemical and functional data, also performed typically on bulk population of samples, is essential as well.

## Electron Tomography of Complex Specimens

While icosahedral viruses and capsids exhibit symmetry and well-defined architecture, many enveloped viruses exhibit pleomorphy and are less singular in organization. In such cases, due to the particle-to-particle variation in morphology, it is not possible to average images of whole viruses to gain more detailed information. Instead, tomography provides a means of gathering three-dimensional structural information by tilting a specimen to image a field of view over a range of angles [2]. This imaging scheme is subject to some basic limitations that result in reduced resolution (on the order of 10 Å resolution) relative to single-particle cryo-EM. Namely, samples are subjected to significantly higher cumulative doses of ionizing radiation as the same region of interest is imaged with tens of repeated exposures during collection of the tilt-series. In addition, mechanical constraints limit a sample’s tilt angle to ~±70°, which leads to incomplete structural information and distortion in the resulting reconstructions. This can be alleviated to some extent using dual-tilt configurations, which allow tilt-series along two orthogonal axes to be gathered, providing more complete data. The necessary instrumentation, however, is not as widely available as the more common single-axis setup. The resulting projection images from the tilt-series are computationally reconstructed to generate the tomographic density. This approach has been applied to image ultrastructure of complex viruses starting with herpes simplex virus-1 [36]. More recently, cryo-electron tomography (cryo-ET) has been applied to characterize paramyxoviruses, influenza viruses, HIV-1, filoviruses, tailed bacteriophages, and numerous other enveloped viruses (see reviews [37,38] and Fig 1). The resolution attainable by cryo-ET is sufficient to distinguish different surface protein types and their conformational states [39–41] as well as to resolve membrane leaflets and the organization of matrix proteins, Gag polyprotein lattices, nucleoprotein complexes, and portal assemblies at unique vertices in otherwise symmetrical capsids. The three-dimensional organization of highly complex, non-symmetrical viruses becomes discernable by cryo-ET.

When repeated features are present in reconstructed density, an averaging approach can be applied to enhance the resolution of the underlying structural features. Sub-tomogram averaging has provided low-resolution structures of glycoproteins in situ on the surface of virions, including HIV-1 Env (reviewed in [42]), Ebola GP [43,44], and influenza hemagglutinin [39,41]. In the case of Ebola GP, tomography was able to localize the heavily glycosylated mucin domain in the context of a complete GP spike, which had been refractory to crystallization [44]. Sub-tomogram averaging was also recently applied to retroviral Gag lattices in immature HIV-1 particles, yielding sub-nanometer resolution reconstructions in which individual α-helices and subunit architecture could be discerned [45]. Notably, rather than being derived from images of recombinant, purified components as in single-particle cryo-EM, the 8.8 Å reconstruction by Schur et al. [45] and the studies of viral surface glycoproteins were based upon imaging of the components within the context of the intact virus or virus-like particles. With further improvements in imaging technology and data analysis, tomography-based structures are likely to increase in resolution. This may at last realize a long-standing goal of extracting high-resolution structures from authentic biological contexts.

## Imaging Dynamic Processes

Cryo-ET can also be employed to gather three-dimensional snapshots of viruses undergoing dynamic processes such as genome injection by bacteriophages [46,47] or membrane remodeling and various stages of fusion by influenza virus [41,48,49], avian sarcoma/leukosis virus [50], HSV-1 [51], and Sindbis virus [52]. Partially mature flavivirus particles imaged by cryo-ET suggested that maturation initiates at the vertices and progresses across the capsid surface [53]. Electron tomography offers perhaps the only means to directly characterize the structures of these types of transient, asymmetrical intermediates under near-native, frozen-hydrated conditions, which is critical for understanding their function.

## Towards the Interface of Structural Virology and Cell Biology

Cell-free virus represents only one stage of the viral infectious cycles. Dynamic events ranging from receptor engagement to uncoating of the genome to replication and assembly take place in the context of the host cell. Cryo-EM is also offering new insights at this level of structural hierarchy. Seminal studies of virus entry into cells in the early 1980s using transmission electron microscopy (TEM) of thin-section, plastic embedded cells (e.g., [54]) provided evidence for endocytic uptake. Such methods, however, are limited in resolution and can suffer in preservation of native cellular structures. Recent studies have used cryo-ET of unfixed specimens to image viruses in cells and tissues. Groundbreaking studies of HSV-1 entry and intracellular trafficking in neurons were among the first to advance structural virology into the realm of cell biology (Fig 1) [51,55]. Enveloped virus assembly at budding sites has been imaged, offering insights into virion morphogenesis (e.g., [56,57]). Interferon-induced antiviral processes against budding HIV-1, mediated by tetherin, have also been imaged recently by cryo-ET, illustrating the interplay of host cell and virus in a native biological context [58]. Lastly, in a cryo-ET study of infected cyanobacteria, assembly intermediates of the cyanophage Syn5 in the intracellular environment of its *Synechococcus* host were imaged at nanometer resolution [59]. This study provided unprecedented glimpses into changes in both phage and host cells resulting from the natural infection.

Because cryo-ET, as a transmission EM approach, relies upon penetration of the electron beam through the specimen, thick specimens often do not yield interpretable images. The use of higher energy electrons or a post-sample energy filter, which removes scattered electrons that would blur the image on the detector [60], helps to some extent, but cellular cryo-ET at present is mostly confined to examining processes such as cell entry and enveloped virus budding that take place at the periphery of cells. Cryo-microtomy, which involves shaving thin slices of unfixed, flash-frozen specimens followed by cryo-ET, enables thick samples to be deconstructed into layers; however, this technically challenging technique is susceptible to the introduction of artifacts and distortion during the preparation of specimens [61,62]. Another approach that has made inroads into analysis of thicker specimens employs a focused ion beam to mill away strata of a thick specimen above and below a layer of interest, which may lie within thicker regions of a cell [61,63]. One can imagine the wealth of information that may become accessible by looking at cryo-preserved specimens in which virus replication and assembly factories lurk deep within the cell body.

In summary, with the breadth of applications spanning from high-resolution structure determination of isolated viral components and intact virions to imaging viruses in the context of infected host cells, cryo-electron microscopy is helping to integrate biological structure with cell biology, providing a structural basis for understanding infection and pathogenesis.
